# OD11 Application Of Natural Language Processing To Predict Final Recommendation Of Brazilian Health Technology Assessment Reports

**DOI:** 10.1017/S0266462324001466

**Published:** 2025-01-07

**Authors:** Marilia Mastrocolla de Almeida Cardoso, Juliana Machado-Rugolo, Lehana Thabane, Naila Camila da Rocha, Abner Barbosa, Juliana Tereza Almeida, Daniel da Silva Pereira Curado, Denis Satoshi Komoda, Silke Anna Theresa Weber, Luis Gustavo Modelli de Andrade

## Abstract

**Introduction:**

Several countries established health technology assessment (HTA) processes to support decision-making. Considering the high volume of submissions processed by HTA agencies, approaches to determine factors associated with the approval would be beneficial. This study aimed to predict the final recommendation of the National Committee for Health Technology Incorporation (Conitec) using a natural language processing (NLP) algorithm for text extraction.

**Methods:**

Conitec’s 2012 to 2022 reports (n=389) were split into 75 percent training and 25 percent testing data. Tokenization enabled NLP models: Least Absolute Shrinkage and Selection Operator (LASSO), logistic regression, support vector machine (SVM), random forest, neural network, and Extreme Gradient Boosting (XGBOOST). Evaluation criteria included accuracy, area under the receiver operating characteristic curve (ROC AUC) score, precision, and recall. Cluster analysis with k-modes identified two clusters (group 0 = approved, group 1 = rejected).

**Results:**

The neural network model demonstrated the best accuracy metrics with a precision of 0.815, accuracy of 0.769, ROC AUC of 0.871, and a recall of 0.746. Some tokenization identified that linguistic markers could contribute to the prediction of incorporation decision by the Brazilian HTA Committee, such as international HTA agencies’ experience and the government as the main requester. Cluster and XGBOOST analysis identified similar results with approved technologies with a predominance of drugs assessment, mainly requested by the government, and not approved mostly assessing drugs, the industry as the main requester.

**Conclusions:**

The NLP model could identify predictors for the final decision process on the incorporation of health technologies in Brazil’s Unified Health System, opening paths for future work using HTA reports coming from other agencies. This model could potentially improve the throughput of HTA systems by supporting experts with prediction/factors/criteria for approval or nonapproval as an earlier step.

